# Non-Oncological Progression of an Aneurysmal Bone Cyst (ABC) in a Puppy Following Surgical Treatment

**DOI:** 10.3390/ani16182856

**Published:** 2026-09-11

**Authors:** Anna Michalska, Jakub Kaczmarek, Daniel Kaup, Annika Lehmbecker, Magdalena Morawska

**Affiliations:** 1 Willows Veterinary Centre and Referral Service, Solihull B90 4NH, UK; michalskavet@gmail.com; 2 Vet Zentrum, 50933 Cologne, Germany; 3 TKE Veterinary Clinic, 66583 Spiesen-Elversberg, Germany; daniel_kaup@web.de; 4 IDEXX Laboratories, 70806 Kornwestheim, Germany; annika-lehmbecker@idexx.com; 5 Department of Surgery and Radiology with Clinic, Faculty of Veterinary Medicine, University of Warmia and Mazury, 10-719 Olsztyn, Poland; magdalena.morawska@uwm.edu.pl

**Keywords:** bone cyst, veterinary surgery, dog, bone grafting, orthopaedic surgery

## Abstract

Aneurysmal bone cysts (ABCs) are benign but locally aggressive osteolytic lesions that are rarely reported in dogs and may progress or recur following surgical treatment. This case report describes the diagnosis, surgical management, postoperative progression, and long-term outcome of an ABC affecting the left ulna of a four-month-old Labrador Retriever. Radiography and computed tomography revealed a rapidly progressive, expansile osteolytic lesion with marked cortical thinning. Histopathological examination supported the diagnosis of ABC, with no evidence of malignancy. Initial treatment consisted of corticotomy, intralesional curettage, autologous bone grafting, and stabilization of the cortical segment with lag screws. Despite complete clinical resolution of lameness, surveillance radiographs six weeks postoperatively demonstrated progression of the lesion proximal to the original surgical site. Revision surgery involving a larger cortical window and more extensive curettage was subsequently performed, followed by bone grafting. Histopathology again confirmed ABC without malignant transformation. The dog regained normal limb function, and serial radiographic and computed tomographic examinations demonstrated progressive osseous remodeling with no evidence of further progression or recurrence through 35 months of follow-up. This case highlights the importance of adequate surgical excision and scheduled postoperative imaging, as ABC progression may occur despite apparent clinical recovery.

## Simple Summary

Aneurysmal bone cysts are rare, non-cancerous bone lesions that can cause extensive bone destruction and may return or continue to grow after treatment. This report describes a four-month-old Labrador Retriever with a rapidly progressive lesion of the left ulna. The diagnosis was established using diagnostic imaging and microscopic examination of bone samples. The dog was initially treated surgically by removing the abnormal tissue and filling the resulting bone defect with bone graft. Although the dog recovered clinically and showed no lameness, follow-up radiographs six weeks later revealed progression of the lesion beyond the original surgical site. A second, more extensive surgery was therefore performed to remove the remaining abnormal tissue. The dog subsequently regained normal limb function, and no further progression or recurrence was detected during 35 months of follow-up. This case demonstrates that apparent clinical recovery does not necessarily indicate complete resolution of a bone lesion. Adequate removal of abnormal tissue and regular postoperative imaging are important for detecting disease progression early, even when the animal shows no clinical signs.

## 1. Introduction

Aneurysmal bone cysts (ABCs) are expansive, benign osteolytic lesions characterized by large vascular sinusoids partitioned by connective tissue septa into blood-filled cavities [1,2,3]. While extensively documented in human medicine, particularly among children and young adults [2,3,4], ABCs are less frequently reported in the veterinary literature. Most cases involve dogs, foals, and cattle [1,3,4,5,6,7,8,9,10]. A single documented case in a young cat has also been reported [11]. The etiology of ABCs remains unclear and involves neoplastic, traumatic, genetic, and hormonal factors [1,7,8,12,13].

ABCs predominantly arise in the metaphyses of long bones [14], yet they have also been reported in other anatomical sites, including the spine, pelvis, short bones, or scapula [5,6]. Although classified as benign, cases of malignant transformation have been documented in humans [15,16] and one dog [6]. Surgical management strategies vary and can include curettage, intralesional excision, or en bloc excision, frequently combined with bone grafting [2,4,7,10,17]. Surgical technique selection is a critical factor influencing the likelihood of recurrence following ABC treatment [2,16,18,19]. Recurrence rates associated with curettage—the method employed in our case—are reported to range from 10 to 30% and are influenced by variables such as age, cyst location, and incomplete excision of cystic material [16,17,19,20].

In humans, numerous reports have described the evolution of ABCs, including spontaneous recurrence and their presumed etiologies. In the veterinary literature, while one case of oncologic recurrence has been reported in a dog 33 months postoperatively [6], to our knowledge there are no documented cases of disease progression despite prior surgical intervention. In this report, progression is defined as radiographic extension of the original, incompletely excised lesion beyond the primary surgical site, whereas recurrence denotes the appearance of a new lesion at a site of previously complete excision. Our report provides evidence that such progression may occur in animals and highlights the importance of employing a sufficiently aggressive yet rational surgical approach, along with regular postoperative monitoring—even in the absence of lameness—to enable early detection and management of disease progression. Definitive disease resolution was achieved following a more extensive second surgery, as confirmed by 35-month follow-up.

## 2. Case Description

### 2.1. Clinical Presentation and Investigation

**Figure 1:**
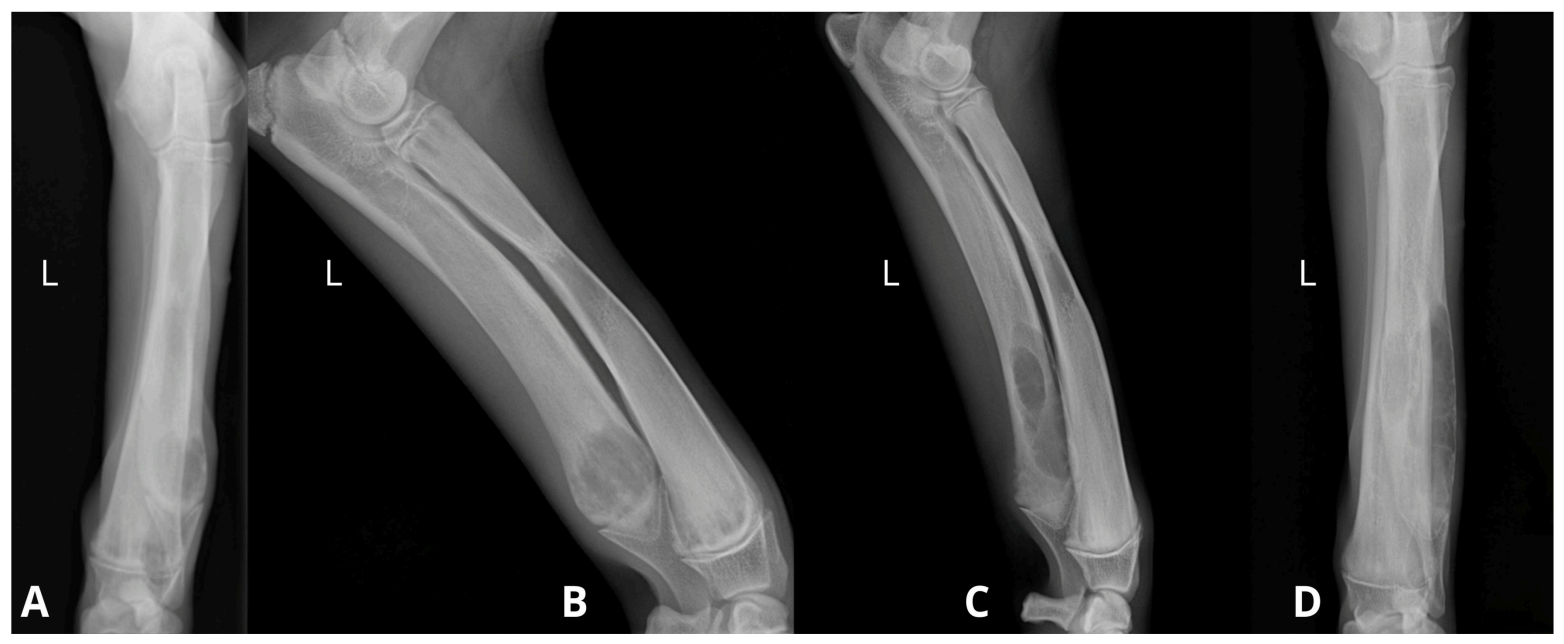
Initial and follow-up radiographic findings in a four-month-old Labrador Retriever with aneurysmal bone cyst (ABC) of the left ulna. (**A**,**B**) Initial craniocaudal and mediolateral radiographs of the left antebrachium. (**C**,**D**) Mediolateral and craniocaudal radiographs obtained three weeks later, demonstrating progression of the osteolytic lesion in the distal ulna with marked cortical thinning and an expansile, multilocular appearance.

A four-month-old, 22 kg male intact Labrador Retriever was presented to the general practice with left thoracic limb lameness and no history of trauma. Physical examination revealed a body temperature of 39.8 °C and marked swelling of the distal left antebrachium, with no other abnormalities. Radiographs of both thoracic limbs (Figure 1A,B) suggested panosteitis. Initial treatment included a two-week course of 16 mg/kg amoxicillin-clavulanic acid every 12 h orally (Clavaseptin, Vétoquinol, Ismaning, Germany), a three-week course of 0.1 mg/kg meloxicam once daily orally (Metacam, Boehringer Ingelheim, Ingelheim am Rhein, Germany), and activity restriction. At the three-week follow-up, clinical signs persisted, and radiographic re-evaluation indicated disease progression (Figure 1C,D); therefore, the dog was referred to a veterinary orthopedic specialist.

During the referral visit, orthopedic examination revealed grade 4/5 lameness [21] of the left thoracic limb, visible and palpable thickening of the lower antebrachium, and minimal external rotation of the limb with carpal valgus, likely indicative of a compensatory postural adaptation to alleviate discomfort during limb weight-bearing. Swelling and pain were noted on the lateral aspect of the left distal ulna, particularly during supination, pronation, carpal flexion and elbow extension. Hematological and serum biochemistry analyses were normal except for an elevated C-reactive protein level (34.4 mg/L; reference range, <10 mg/L).

**Figure 2:**
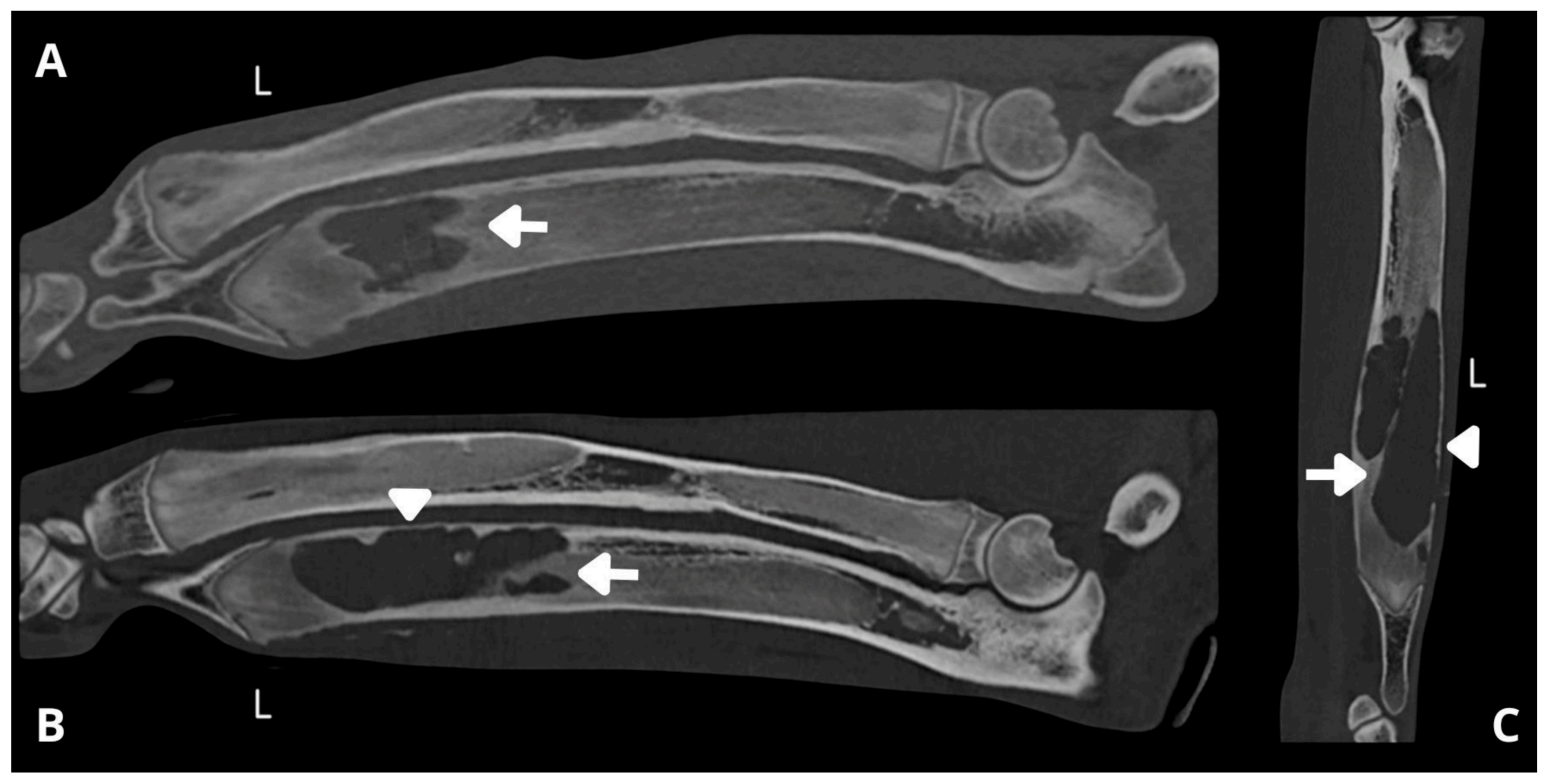
Computed tomography findings of the left thoracic limb in a four-month-old Labrador Retriever with aneurysmal bone cyst (ABC). (**A**) Sagittal CT image showing the expansive osteolytic lesion involving the distal ulna (arrow). (**B**) Follow-up sagittal CT image demonstrating marked progression of the osteolytic lesion (arrow), with cortical thinning (arrow head) and a multilocular appearance. (**C**) Dorsal CT reconstruction showing the extent of the lesion within the distal ulna (arrow) and associated cortical destruction (arrow head).

The dog underwent computed tomography (CT) imaging of both thoracic limbs and the thoracic cavity under general anesthesia, which suggested the coexistence of several conditions (Figure 2A). Distinct areas of sclerosis were visible within the marrow cavities of the left humerus, left radius, and ulna bilaterally. Panosteitis was initially considered because of the patient’s young age, large breed and presenting lameness, and the patchy osteolytic lesions observed in the metaphysis of the right radius. In the left ulna, osteolysis extended from the distal metaphysis to the proximal diaphysis, involving 80% of the bone’s diameter (lesion length: 27 mm; cortical thickness: 3 mm). The “moth-eaten” osteolytic changes in the metaphyses suggested metaphyseal osteopathy (hypertrophic osteodystrophy). The aggressive appearance of the osteolytic lesions favored a diagnosis of hematogenous metaphyseal osteomyelitis. Additionally, the expansive osteolytic lesion in the distal ulna raised suspicion of fibrous dysplasia as a possible concurrent condition.

Further diagnostics, including bone biopsy for histopathological, bacteriological, and mycological analyses, as well as blood culture, were advised but declined by the owner due to financial constraints. Given the increased risk of pathological fracture in the affected ulnar region, activity restriction was strongly advised, 0.1 mg/kg meloxicam once daily orally was recommended for continued analgesic therapy, and a follow-up evaluation was scheduled in three weeks.

On scheduled re-evaluation, the dog exhibited partial weight-bearing standing and non-weight-bearing walking lameness, mild left carpal valgus, moderate tenderness over the left ulna, and normal range of motion in both thoracic limbs. Follow-up CT (Figure 2B,C) revealed that sclerotic changes in the left humerus, left radius, and ulnae bilaterally remained broadly stable. However, a marked advancement of expansive osteolytic changes, an osteolytic bone lesion surrounded by ballooned periosteum, and a multilocular arrangement was noted in the left ulna, indicating disease progression (lesion length: 61 mm, an increase of 126% from the initial CT; cortical thickness: 0.9 mm). The cortical bone was markedly thinned and exhibited endosteal scalloping. Severe cortical thinning led to concern for pathologic fracture, prompting a recommendation and consent from the owner for bone biopsy.

**Figure 3:**
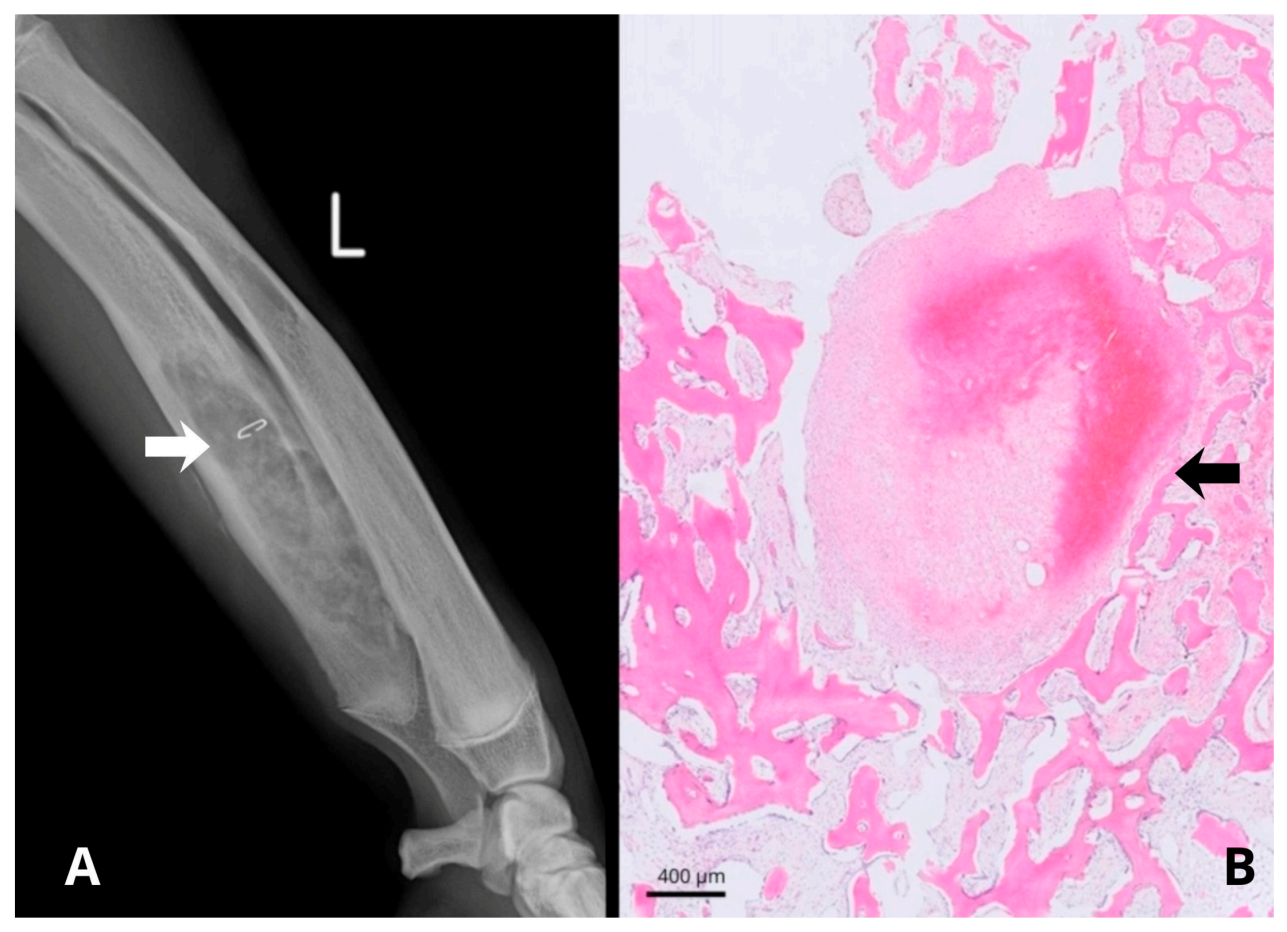
Histopathological findings, surgical treatment, and early postoperative radiographic appearance of the aneurysmal bone cyst (ABC) of the left ulna. (**A**) Radiograph of the left antebrachium following core needle biopsy (arrow). (**B**) Histopathological section showing multinucleated giant cells (osteoclasts, arrow) within connective tissue and adjacent bone trabeculae (H&E; scale bar = 400 μm).

Under general anesthesia, following aseptic preparation of the left antebrachium, a core needle biopsy of the left ulna was performed using an 18G × 7.9 cm T-handle Jamshidi needle (Argon Medical, Plano, TX, USA) (Figure 3A) [22]. Five tissue specimens were collected for histopathological, bacteriological, and mycological analysis. Mycological and bacterial cultures were negative. Histopathological examination of the submitted tissues revealed collagen-rich connective tissue with moderate cellularity and scattered pigment-containing macrophages. Additionally, one fragment contained both cartilage and trabecular bone; the trabeculae were thin and covered by a single layer of osteoblasts embedded in loose connective tissue. A few samples displayed focal areas devoid of osteoid with evidence of hemorrhage. Among the trabeculae, there were regions densely populated with multinucleated giant cells (osteoclasts), macrophages, and the occasional neutrophil (Figure 3B). No signs of malignancy were detected. Notably, the absence of the abnormal osteoid matrix production pattern characteristic of fibrous dysplasia, together with the lack of atypical mitotic figures and malignant osteoid formation seen in telangiectatic osteosarcoma, helped exclude these two principal histological mimics. The clinical presentation, preoperative diagnostic imaging, intraoperative macroscopic appearance, and histopathological findings supported a diagnosis of ABC.

### 2.2. Surgical Treatment and Outcome

The left ulna, left proximal humerus, and left ilium were clipped and aseptically prepared. The surgical procedure employed a lateral approach to the left ulna and involved lateral corticotomy and curettage of the bone lesion. On the distal aspect of the lateral cortex of the ulna directly over the lesion, an approximately 6 × 2 cm bone window was created with an oscillating saw. The bone segment was thoroughly curetted, and tissue samples collected from the bone window were sent for histopathological analysis, as well as for both aerobic and anaerobic cultures. The bone cavity was filled with an autologous cortico-cancellous bone graft harvested from the ipsilateral proximal humerus and iliac crest. The ulnar segment was anatomically secured with three 3.5 mm cortical lag screws, and the wound was closed in a regular manner. The left thoracic limb was protected with a modified Robert Jones dressing. Postoperative care included meloxicam (0.1 mg/kg once daily orally), dressing changes every 3–4 days until suture removal after 2 weeks, and physical activity restriction. Radiographic follow-up was recommended at 3 and 6 weeks postoperatively.

**Figure 4:**
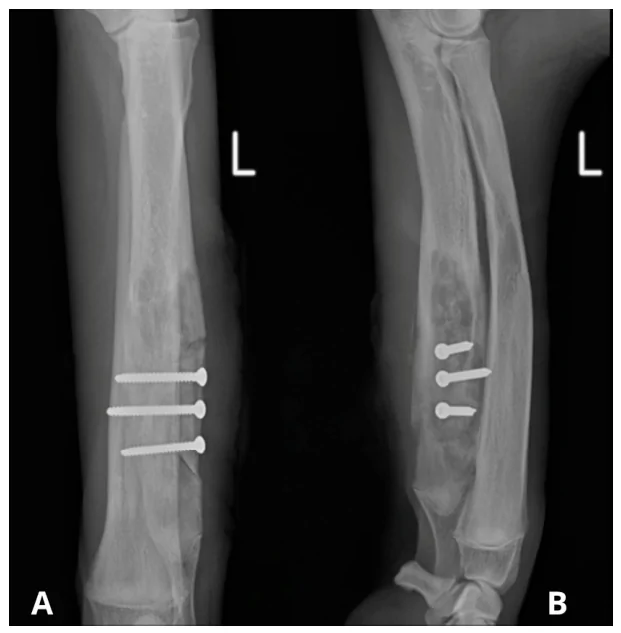
(**A**,**B**) Craniocaudal and mediolateral radiographs obtained three weeks after curettage, autologous bone grafting, and stabilization of the cortical window with three cortical lag screws, showing the early postoperative appearance without apparent complications.

Three weeks post-surgery, clinical assessment revealed no lameness or pain; therefore, the dog no longer required oral analgesics. Radiographs at 3 weeks showed no apparent complications (Figure 4A,B).

**Figure 5:**
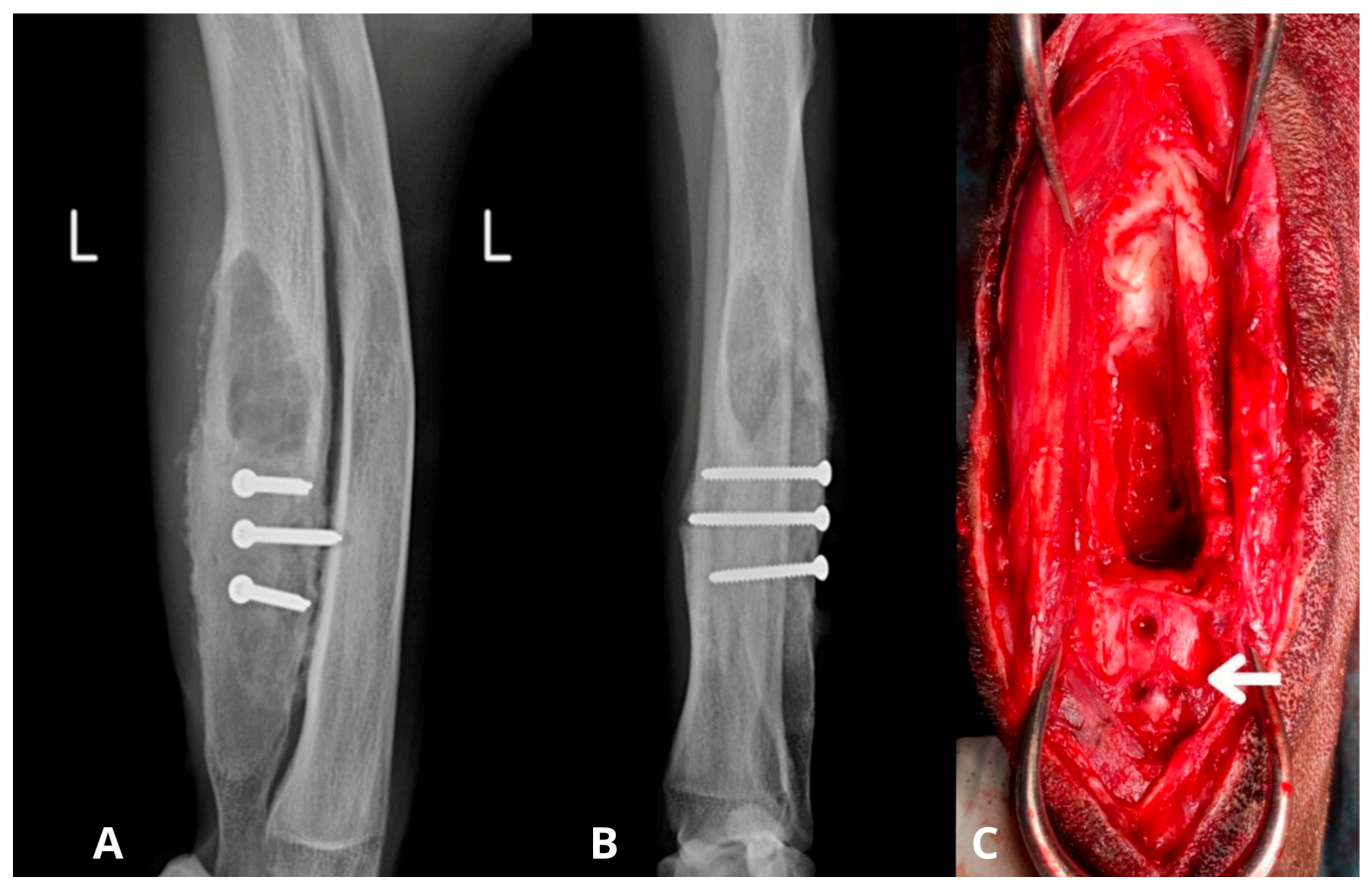
Radiographic evidence of aneurysmal bone cyst progression and intraoperative findings during revision surgery. (**A**,**B**) Mediolateral and craniocaudal radiographs of the left ulna obtained six weeks after the first surgery, showing progression of the cystic lesion proximal to the original surgical site. (**C**) Intraoperative view during revision surgery following extension of the cortical window and curettage of the recurrent/progressive lesion; the arrow indicates the original distal cortical window.

Six weeks post-surgery, both general and orthopedic examinations were unremarkable. However, repeat standard radiographic evaluation of the left thoracic limb revealed a suspicious area of cyst progression just proximal to the original surgical site (Figure 5A,B) (new lesion length: 43 mm; estimated 72% of the original, surgically created cortical window). Consequently, a decision was made to proceed with a second surgery. Under general anesthesia, a lateral approach to the left ulna was performed, the cortical screws were removed, and an extended cortical window (2 cm × 8 cm) was created proximal to the initial window to access the new lesion (Figure 5C). The medullary cavity was curetted more aggressively using a curette and high-speed burr, then filled with cancellous bone graft harvested from the contralateral (right) iliac crest and humerus. The contralateral side was selected because the ipsilateral proximal humerus and iliac crest had already been used as donor sites during the first surgery and were still healing; harvesting fresh, undisturbed cancellous bone from the opposite side avoided disrupting the previously grafted donor sites and provided a more reliable volume of good-quality graft material, while also reducing cumulative donor-site morbidity. Haemostasis was again achieved with bone wax. The original cortical segment was reattached and secured with three lag screws. The surgical site was closed routinely, and a modified Robert Jones bandage was applied. Histopathological evaluation of the debrided tissue from this second surgery again confirmed ABC with no evidence of malignancy. Postoperative management included meloxicam (0.1 mg/kg PO once daily) for 7 days, bandage changes every 3 days until suture removal at 14 days, and strict activity limitation for 12 weeks followed by a gradual return to normal activity.

**Figure 6:**
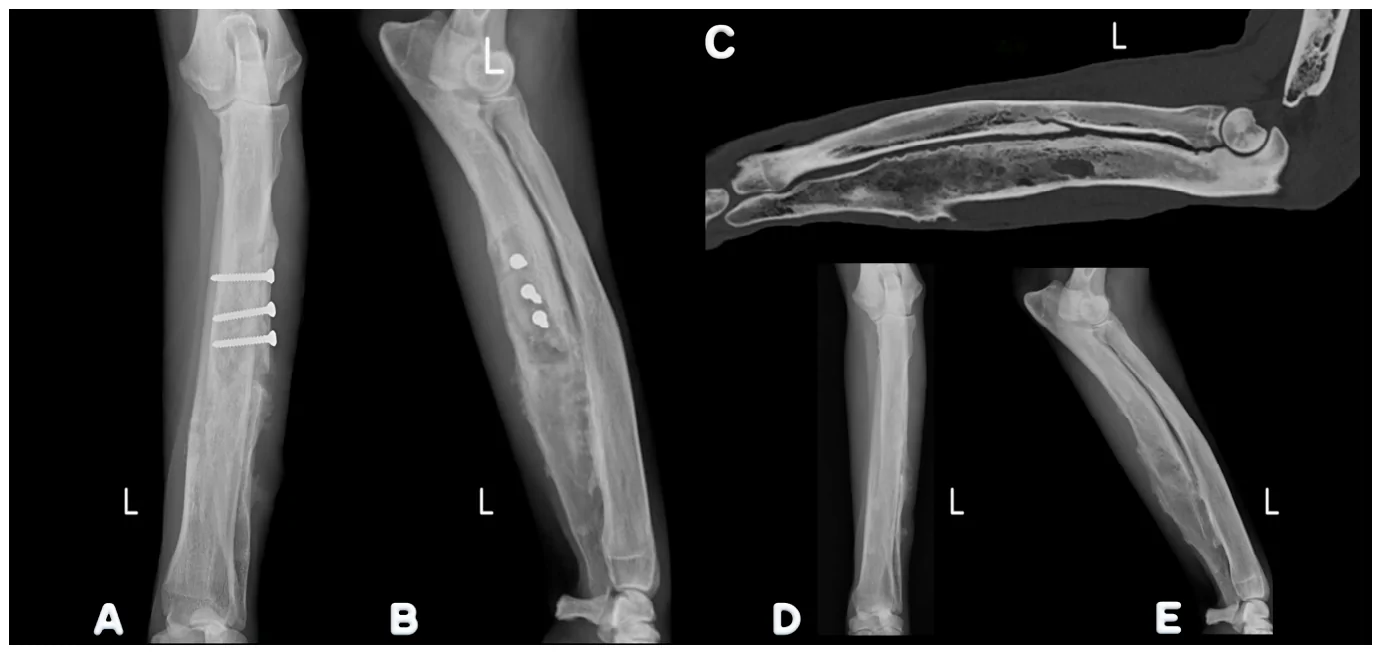
Radiographic and computed tomographic follow-up after revision surgery for an aneurysmal bone cyst (ABC) of the left ulna. (**A**,**B**) Craniocaudal and mediolateral radiographs obtained eight weeks after the second surgery, showing progressive bone healing without further expansion of the cystic lesion. (**C**) Sagittal CT image obtained 10 months after the second surgery, following removal of the cortical screws, demonstrating bone remodeling and no evidence of cyst recurrence. (**D**,**E**) Craniocaudal and mediolateral radiographs obtained 24 months after the second surgery, showing advanced osseous remodeling and no radiographic evidence of recurrence.

Follow-up examinations at 4 and 8 weeks postoperatively demonstrated progressive resolution of lameness, and radiographs of the left ulna showed no further expansion of the bone cyst (Figure 6A,B).

The dog was scheduled for a re-check 6 months postoperatively; however, the owner presented him at 10 months instead, due to the absence of clinical concerns and personal commitments. There was no lameness reported, but orthopedic examination elicited mild pain on palpation over the lateral ulna at the screw sites. The screws were electively removed, and the postoperative CT confirmed the absence of the recurrent cyst (cortical thickness at the graft site: between 1.1 and 1.8 mm) (Figure 6C).

Given the need for long-term monitoring in patients diagnosed with ABC [10], the owner was advised to return for annual orthopedic evaluations (or sooner if any clinical signs arose). At routine follow-up examinations 24 and 35 months after the second surgery, the dog had regained full and normal use of the limb. Orthopedic exams were unremarkable, and radiographic assessments confirmed no evidence of cyst recurrence at 24 months (Figure 6D,E) and at 35 months postoperatively.

## 3. Discussion

A range of medical and surgical management strategies for ABCs has been documented, with healing outcomes and recurrence rates varying depending on the method employed. Surgical procedures—particularly curettage combined with cancellous bone grafting, as applied in our case—have been associated with a high rate of healing, with success reported in up to 87% of cases, compared to approximately 51% for non-surgical treatments, such as percutaneous sclerotherapy, arterial embolization, and radiotherapy [11,21,23]. It is widely recognized that combining intralesional curettage with autologous cancellous bone grafting offers several biological advantages, including the provision of an osteoconductive scaffold, osteoinductive growth factors, and osteogenic cells—elements essential for effective bone regeneration [24]. Nevertheless, given the inherent risk of recurrence in aneurysmal bone cysts, even an initially favorable postoperative outcome should be interpreted with caution. A structured and consistent follow-up protocol is crucial for the early detection and management of disease progression, as exemplified in this case. The progression observed was most likely related to an inadequately sized cortical window and incomplete removal of the cystic tissue (the initial cortical window was estimated to cover approximately 72% of the total lesion length on retrospective comparison with preoperative CT). This interpretation is further supported by the definitive and sustained resolution achieved following a more extensive and aggressive surgical approach during the second procedure. Furthermore, many aneurysmal bone cysts develop near open physes or articular cartilage in skeletally immature patients [25]. Concern over damaging these critical structures may lead surgeons to adopt a less aggressive curettage approach (as may have occurred in our case). While complete excision of the lesion is essential to minimize recurrence risk, surgical aggressiveness must be balanced to avoid iatrogenic damage to a physis or articular surface, which can result in permanent limb length discrepancies or joint dysfunction [26].

ABCs have been reported to develop at sites of previous trauma, as primary bone lesions, or secondary to other osseous tumors such as giant cell tumors, chondroblastomas, osteoblastomas, or other skeletal abnormalities [2,20,22]. Although ABCs are classified as benign, malignant transformation has been documented not only in humans [8,27] but also in a dog [6]. In the latter, a previously reported case involving a Labrador Retriever described recurrence of an ABC 33 months postoperatively, presenting with acute-onset lameness; histopathology at the time of recurrence confirmed the presence of a chondrosarcoma. In contrast, in our case, a preoperative biopsy and two postoperative histopathological evaluations revealed no evidence of neoplastic transformation, and the dog remained clinically sound and free of lameness for 35 months following the second surgery. Nevertheless, it should be emphasized that, even though our follow-up period exceeded the 33-month interval reported in that earlier case, the possibility of future malignant transformation cannot be completely excluded—particularly in light of evidence suggesting that surgical manipulation of ABCs may predispose to such changes [6,15]. Moreover, considering that radiographic assessment alone may fail to identify early malignant transformation [6,26,28,29], it is recommended that any recurrence of lameness in similar cases be promptly investigated using advanced imaging modalities (e.g., CT), followed by biopsy and histopathological examination when indicated.

In this case, routine postoperative radiographic evaluations at 3 and 6 weeks enabled early detection of disease progression despite the absence of lameness and an otherwise unremarkable clinical examination. It should be emphasized that a seemingly normal early postoperative presentation in a dog that has undergone orthopedic surgery—particularly when some degree of lameness would normally be expected—may mask the true status of bone healing, potentially leading to an overly optimistic assessment of outcome. Therefore, clinical findings should always be corroborated with scheduled radiological follow-ups, which should not be omitted even in the early postoperative period, as demonstrated in this case.

A human study evaluating various imaging modalities for ABC diagnosis showed that MRI and CT are comparably effective (diagnostic accuracy about 75% and 63%, respectively), even in challenging cases, and that accuracy improves significantly when multiple imaging techniques are combined [28]. In our case, radiographs and CT were the chosen imaging modalities. Although adding MRI would likely not have altered our clinical decision-making, it might have provided additional diagnostic detail and educational value.

A key limitation of the present report lies in its retrospective nature. The analysis relied exclusively on previously collected clinical data, radiographs, and CT images, without a predefined follow-up schedule. Consequently, subtle radiological changes between the 10- and 35-month follow-ups may have gone undetected, and no histopathological reassessment was performed at the final visit to confirm the persistence of benign pathology. Furthermore, as with most retrospective case reports, owner information regarding postoperative activity restriction and owner compliance was limited and could not be objectively verified.

## 4. Conclusions

This case report underscores the importance of achieving an appropriate degree of surgical aggressiveness to minimize the risk of ABC progression—a principle consistent with evidence from the human medical literature [8]. It should be remembered that although ABCs frequently develop adjacent to open physes or articular cartilage in skeletally immature patients, raising legitimate concern about potential iatrogenic damage during curettage, complete excision of the lesion remains essential to minimize the risk of recurrence. Striking an appropriate balance between sufficient surgical radicality and the preservation of critical growth and joint structures is therefore fundamental to achieving successful long-term outcomes. It also highlights the critical role of routine postoperative clinical and radiological assessments in the early detection of ABC progression, which may occur despite an initially benign clinical presentation. Such diligence in follow-up enables timely intervention and improves the likelihood of complete disease resolution.

## Data Availability

The data supporting the findings of this case report are available from the corresponding author upon reasonable request.
